# The emerging importance of METTL5-mediated ribosomal RNA methylation

**DOI:** 10.1038/s12276-022-00869-y

**Published:** 2022-10-21

**Authors:** Elena M. Turkalj, Caroline Vissers

**Affiliations:** 1 Developmental and Stem Cell Biology Graduate Program, University of California San Francisco, San Francisco, CA 94107 USA; 2 Department of Biochemistry & Biophysics, University of California San Francisco, San Francisco, CA 94107 USA

**Keywords:** RNA metabolism, Mechanisms of disease, Neurological disorders

## Abstract

The study of the epitranscriptome has thus far focused largely on mRNA methylation. Recent human genetics studies suggest that methylation of ribosomal RNA also contributes to brain development and cognition. In particular, the m^6^A modification at the A-1832 position of the 18S rRNA is installed by METTL5. Mutations or deletions of Mettl5 in humans and mice, respectively, cause abnormal translation and gene expression that in turn mediates stem cell behaviors such as differentiation. In this review, we provide an overview of the current knowledge of the methyltransferase METTL5, as well as the molecular biology surrounding m^6^A on rRNA and how it regulates cell behavior.

## Brain development: the role of ribosomal RNA modification

A specific modification of ribosomal RNA (rRNA) may contribute to intellectual disability and cognitive dysfunction. All RNA molecules, including rRNA, are biochemically modified after transcription. Modifications can be influenced by external factors and can affect gene expression, although the exact mechanisms remain unclear. Elena Turkalj and Caroline Vissers at the University of California San Francisco, USA, reviewed current understanding of an rRNA modification called m^6^A_1832_ carried out by METTL5, a methyltransferase enzyme that plays a significant role in early development. Recent studies suggest that methylation of rRNA, including the m^6^A_1832_ modification, directly influences brain development and cognition. *METTL5* gene mutations are linked with intellectual disabilities. Future work should examine how m^6^A_1832_ modifications vary under different biological and environmental conditions, and how they are linked to *METTL5* expression.

## Introduction

The field of epitranscriptomics has garnered major interest in the past 10 years as the substrates of various methyltransferases have been identified. While hundreds of different modifications are known to be present on RNAs, the exact functions and mechanisms of action for most modifications remain unclear. Furthermore, a major hurdle in the field has been developing high-resolution methods for the identification and quantification of RNA modifications, which is generally necessary to elucidate their functions. In particular, the study of methylation at the N6 position of adenosine on mRNA, termed m^6^A, has grown rapidly due to improvements in detection. m^6^A on mRNA is especially interesting because it is highly dynamic—methylation and demethylation change in response to various stimuli—and because m^6^A can be added to many different gene transcripts in a context-dependent manner^1,2^. This modification allows for transcript-specific regulation, as modified transcripts are individually processed by m^6^A reader proteins that alter their processing within the cell^3,4^. Since m^6^A on mRNA has been heavily studied over the past decade, many reviews are currently available^5–9^.

A much more recent topic of interest in the field of epitranscriptomics is m^6^A on ribosomal RNA (rRNA). In particular, *Mettl5* has been identified as the methyltransferase that adds m^6^A to a specific site, nucleotide A-1832 of 18S rRNA, hereafter referred to as m^6^A_1832_^10^. Mutations in the *METTL5* gene in humans cause intellectual disability and developmental abnormalities, and studies in model organisms further suggest a role in development^11–14^. In the past two years, a few key studies have furthered our understanding of METTL5 in terms of its biochemical properties and its function as a regulator of translation^10,13–19^. In this review, we discuss the current knowledge of METTL5-mediated m^6^A_1832_ and its growing importance in the field of epitranscriptomics.

## Biochemical and biophysical properties of METTL5

### Binding substrates and concentration of m^6^A_1832_

**Fig. 1:**
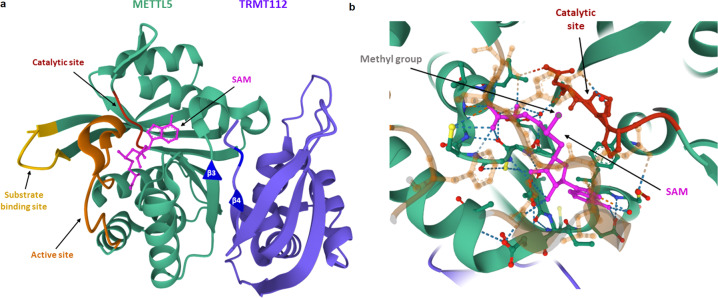
Crystal structure of the human METTL5-TRMT112 complex (PDB code 6H2U). **a** METTL5 and TRMT112 proteins are shown here in turquoise and purple, respectively. The catalytic site of METTL5 is shown in red, the substrate binding site is shown in yellow, and the active site is shown in orange. S-adenosylmethionine (SAM) is shown in hot pink. METTL5 β3 and TRMT112 β4 are shown in dark blue. **b** Zoomed-in view of the interaction between the catalytic site of METTL5 and SAM. 18 S rRNA is not shown. The color scheme is the same as in A except that the methyl group is shown in gray. The catalytic site, SAM, and its methyl group are shown in ball and stick representation. The dotted lines indicate hydrogen bonds.

The m^6^A_1832_ modification on human 18S rRNA was first discovered over 30 years ago^20^, but it was only recently, with technical advances in sequencing, LCMS, and cryo-EM, that METTL5 was identified as the methyltransferase that catalyzes this reaction^10,16^. METTL5 contains a single class I methyltransferase domain with a canonical Rossman fold, a central seven-stranded β-sheet sandwiched by alpha helices with four on one side and two on the other^10^. It contains an active site (V16-T31), a substrate binding site (D185-D199), and a catalytic site (N126-F129)^10,21^ (Fig. 1a, b)^10,22^. Like other N6-adenosine methyltransferases, the METTL5 catalytic site contains a canonical NPPF motif^10^. METTL5 interacts with its substrate, S-adenosyl-L-methionine (SAM), at the C-terminal region of the METTL5 β-sheet to catalyze the addition of the methyl group of SAM at the N6 position of adenosine^10,16^. This results in ^an m6^A_1832_ modification at the 3' minor end of 18S. This is a few bases away from the decoding center—the site where tRNA anticodons interact with the mRNA codon during translation^10,23^. The localization of m6A_1832_ in the decoding center has implications for translation efficiency, which will be discussed later in this review.

Van Tran et al. found that METTL5 heterodimerizes with its coactivator, tRNA methyltransferase subunit 11-2 (TRMT112)^10^. This complex forms a large hydrophobic core and a parallel β-zipper, which is structurally conserved between TRMT112 and its other interacting partners^10,24^. Crystallization of the METTL5-TRMT112 complex revealed that they form a conserved binding area around SAM. Based on previous findings that TRMT112 stabilizes the β3-β4 loop with its partner, it is predicted that TRMT112 strengthens the binding of METTL5 to SAM^22,25,26^ (Fig. 1a)^10,22^. Similarly, METTL5 β3 was found to interact with TRMT112 β4, but the effect of this interaction on SAM binding affinity needs further investigation. Despite the need for additional information on the mechanism, follow-up experiments have confirmed that TRMT112 is important for the metabolic stability and activity of METTL5^10^. Knockdown of TRMT112 led to reduced protein levels of METTL5, and coexpression of METTL5 and TRMT112 significantly increased the methyltransferase activity on 18S rRNA^10,15,19^. This finding indicates that TRMT112 is not required for METTL5 catalytic activity but significantly amplifies and stabilizes it.

Next, Ignatova et al. found that METTL5 interacts with RNAs >200 nt and with double-stranded RNA^16^. Upon further investigation, they confirmed that METTL5 catalyzes the m^6^A_1832_ modification of 18 S rRNA, but they did not specify whether the 18 S substrate was in single-stranded (immature) or double-stranded (mature) form. Sendic et al. found that METTL5 modifies a conserved rRNA UAACA motif in vitro, at adenosine 1832 in humans and adenosine 1717 in *C. elegans*^27^. Rong et al. and Sendic et al. found that the length of the rRNA sequence contributes to methylation efficiency, with an 11 bp probe resulting in higher levels of METTL5 activity compared to other probe lengths^18,27^. Notably, METTL5 was found to interact with the sequence motif only on single-stranded probes^27^. This finding indicates that METTL5 likely interacts with 18S during rRNA processing before the A1832 residue is in the mature double-stranded form^10,27^. Although METTL5 interacts with the 18S motif in its single-stranded form, its interaction with double-stranded RNAs and polyadenylated RNAs raises the possibility that METTL5 may also have other substrates^16,19^. METTL5 is localized in the nucleolus and cytoplasm, but it is not clear where it interacts with its substrates. Future studies are necessary to investigate the timing, location, and mechanisms of METTL5 interactions with substrates.

rRNA is highly abundant in the cell and makes up ~80% of the RNA in mammalian cells, and ~2% of all rRNA nucleotides are chemically modified^28–31^. Although most 18S rRNAs are m^6^A_1832_ modified, there is variability in m^6^A_1832_ abundance between cell types and organisms. This variance could suggest that m^6^A_1832_ is dynamic and provides a regulatory function in response to stimuli, but no m^6^A_1832_ demethylase has been identified, and the 3' end of the 18S rRNA is consistently highly methylated. In human cell lines such as TK6 and HeLa, ~99–100% of rRNA has the m^6^A_1832_ modification^32^. In other cell types, such as mESCs, rRNA A1832 was found to be methylated 60 to 70% of the time^16^, while *C. elegans* rRNA is m^6^A modified at A1832 98% of the time^33^. Similar to the m^6^A modifications on mRNA, 18S rRNA m^6^A modifications may be more dynamic under different biological conditions^34,35^. A single paper showed that in HEK293T, HeLa, and 3T3 cells, m^6^A_1832_ levels are higher under sulfur and methionine starvation conditions than under normal conditions, indicating that different environmental conditions can cause variability in the abundance of m^6^A modifications of rRNA^36^. Despite the indications of dynamic regulation, further work is needed to find a potential demethylase or a demethylating mechanism. Whether dynamics in m^6^A_1832_ abundance based solely on the expression level and activity of METTL5 are sufficient to regulate the cellular response to stimuli also requires further investigation.

### Tissue and cellular location of METTL5

**Fig. 2:**
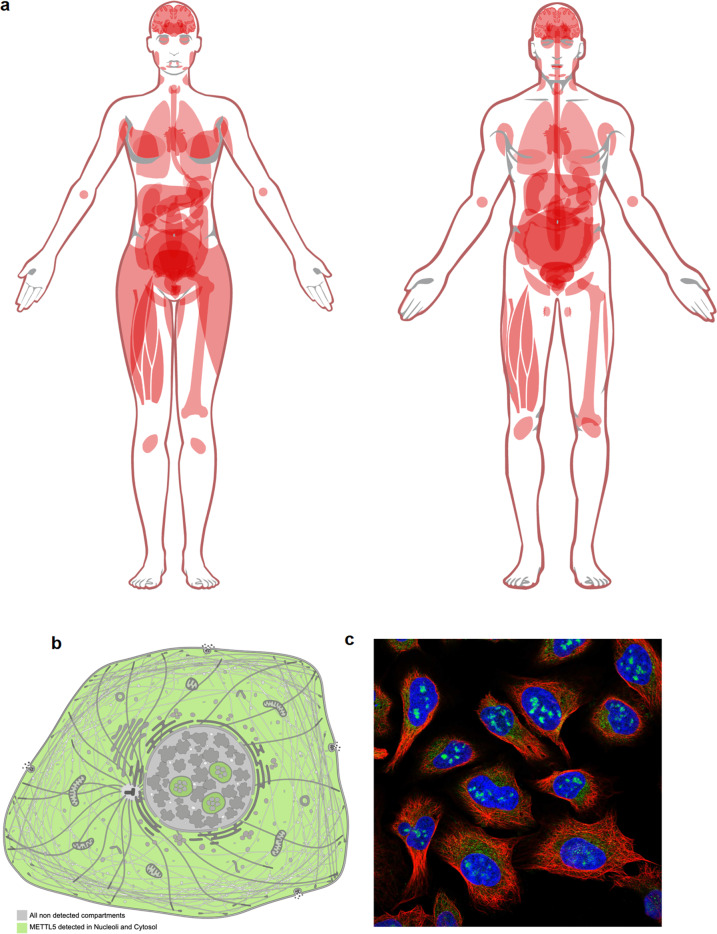
Expression profile of METTL5 according to the Human Protein Atlas. **a** Expression of METTL5 across tissues (image from the Human Protein Atlas: https://www.proteinatlas.org/ENSG00000138382-METTL5/tissue). **b**, **c** The subcellular expression profile of METTL5 shows its localization to nucleoli and cytoplasm (images from the Human Protein Atlas: https://www.proteinatlas.org/ENSG00000138382-METTL5/subcellular).

The functional role of METTL5 may be informed by its location across tissues and within a cell. METTL5 is found to have low but ubiquitous expression across all tissues throughout the adult human body, with the highest expression in the digestive tract, according to the Human Protein Atlas project (Fig. 2a)^37–39^(proteinatlas.org). Most of the tissues tested were from adults aged 40 and over, which showcases the protein expression levels during the later stages of adulthood. Follow-up studies are necessary to verify the METTL5 expression level and location in these tissues. Nevertheless, some studies have indicated that METTL5 expression decreases as tissues age. Rong et al. examined METTL5 expression in tissues from 20- to 70-year-old patients, including brain, muscle, blood, heart, colon, and lung tissues^18^. They identified a significant reduction in the protein expression of METTL5 with age, which suggests that METTL5 may have higher expression in younger tissues and potentially even higher levels in early development^18^. Lower METTL5 protein expression may lead to lower levels of modified 18S rRNA in aged tissues and higher levels in younger tissues, but the direct correlation between METTL5 expression and m^6^A_1832_ concentration needs to be tested.

Despite the lack of METTL5 expression quantification in many young tissues, METTL5 appears to have a significant role in the brain during early development. This is supported by the finding that METTL5 mutations have a profound effect on brain development and intellectual disability^11–13,40^. METTL5 expression in the human brain was found in the cerebellar cortex, hippocampus, and striatum from early development (8 post conception weeks; PCW) to the later stages of adulthood, but whether the protein levels are significantly higher during development than during adulthood remains unquantified^13^. Additionally, postnatal day 30 (P30) mice have low but ubiquitous METTL5 expression throughout the brain, similar to the expression profile found in adult humans^13^. In parallel, the METTL5 binding partner TRMT112 is found to be highly and ubiquitously expressed in rodent brains during embryonic development, suggesting that even low levels of METTL5 are likely stabilized and active^41^.

In cultured rat hippocampal neurons, METTL5 expression was observed in the nucleus and neuronal dendrites^13^. Nuclear localization was also found in other mammalian cell lines, such as COS-7^13^, HEK293Ts^18^, and U2-OS^38^. More specifically, METTL5 tagged with a fluorescence marker was found to localize predominantly in the nucleolus, with some diffuse expression in the cytoplasmic region (Fig. 2b, c)^15,18,19,42^ (proteinatlas.org). Interestingly, HeLa and HepG2 cells were found to have a similar subcellular location profile as other mammalian cell lines, but after quantifying the amount of protein in the cytoplasm and nucleus, it was discovered that METTL5 is predominantly expressed in the cytoplasm^19^. This indicates that METTL5 may change its location over time or that certain cell types have nonstandard METTL5 localization. In Drosophila S2R + cells, Mettl5 protein was also found mostly in the cytoplasm with some minor localization in the nucleolus, even though its coactivator, Trmt112, was found mainly in the nucleolus^17^. The variability in localization suggests that METTL5 may have functionally different roles across cell types or that its activity and dynamics may vary according to its localization within the cell.

### Function of 18S rRNA m^6^A methylation

The exact mechanism of 18S rRNA m^6^A_1832_ methylation as a regulator of translation is unknown, although multiple hypotheses have been reported. Van Tran et al. showed that METTL5 is stabilized by TRMT112 but that loss of METTL5 does not affect cell growth, ribosome biogenesis, or mature rRNA production in HCT116 p53-positive carcinoma cells^10^. The study compared the structure surrounding A1832 between precursor and mature forms of 18S rRNA and found that the immature subunit was in a stretched and more relaxed state. This open conformation is hypothesized to allow more accessibility, enabling the METTL5-TRMT112 complex to methylate A1832^10^. This work was especially important for defining the position at which METTL5 confers m^6^A methylation. More specifically, A1832 is close to the h44-h45 loops that make up the decoding center in the small ribosomal subunit. It is therefore likely that the methyl group contributes to translation in a manner specific to the decoding center.

**Fig. 3:**
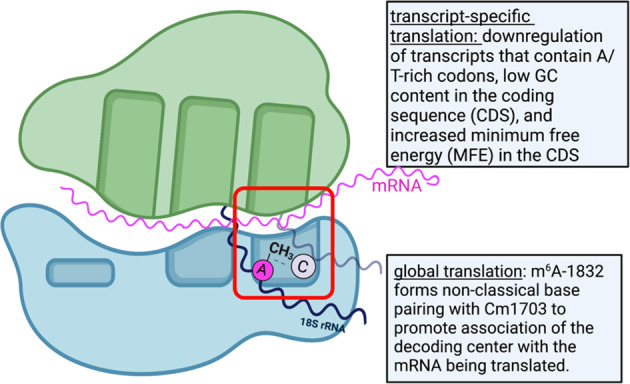
Localization of m^6^A_1832_ in the decoding center of the ribosome suggests a role in regulation of translation. m6A_1832_ methylation in the decoding center (indicated by the red box) of the ribosome could regulate translation in a transcript-specific manner by changing binding affinity of specific mRNAs. Alternatively, m^6^A_1832_ could affect global translation by altering the decoding center structure to more closely associate with many different mRNAs.

Next, Rong et al. hypothesized that m^6^A_1832_ is involved in fine-tuning the structural confirmation of the decoding center to promote translation^18^. Chen et al. and Rong et al. found that m^6^A_1832_ engages in nonclassical base pairing with another nearby rRNA modification, Cm1703, in the h44-h45 loops^15,18^ (Fig. 3). This interaction may cause a conformational change to bring the rRNA of the decoding center closer to the mRNA substrate. Based on this finding, these authors propose that m^6^A_1832_ is involved in fine-tuning ribosome activity by shaping the decoding center toward an mRNA-bound conformation^18^.

### Global vs. transcript-specific translation

Despite the consensus that METTL5 is involved in the regulation of translation, the exact mechanism—and particularly whether it regulates global or transcript-specific translation—is unclear. The high (~99%) abundance of m^6^A_1832_ in human 18S rRNA logically leads to the assumption that it modulates translation at a global scale^32^. However, compounding factors such as ribosome heterogeneity and differential binding affinity of mRNAs create the potential for transcript-specific effects. Along these lines, some rRNA modifications, such as pseudouridine and 2′-O-methylation, are important for translating certain subsets of mRNA by altering ribosomal affinity for those mRNAs^43–45^. Furthermore, despite the ubiquitous nature of m^6^A_1832_, its abundance is dynamic in response to stimuli such as sulfur and methionine starvation^36^, suggesting a regulatory role in the cellular stress response. To date, different studies have identified both transcript-specific and global changes in translation in *METTL5* knockout cell lines^15,18^. Whether these findings are conflicting or simply context-dependent remains to be determined.

Chen et al. found no differences in global translation between *METTL5* KO HEK293T cells and control cells, indicating that METTL5 methylation of 18S rRNA does not have an effect on ribosomal biogenesis or global translation activity^15^. The authors hypothesized that m^6^A_1832_ may be involved in fine-tuning translation of specific RNAs or under specific biological conditions. A study in a mouse B16 melanoma *METTL5* KO cell line showed that a specific subset of genes is downregulated at the translation level; these genes were enriched for Gene Ontology (GO) terms related to cellular stress response. The exact mechanism of action is not yet defined; many regulatory networks change in response to cellular stress, and how METTL5-mediated rRNA methylation fits into the broader cellular response is unclear. Nonetheless, this work supports the hypothesis that 18S methylation of rRNA leads to transcript-specific changes in translation.

Two studies also identified transcript-specific changes in translation when comparing *METTL5* KO and control mESCs. First, Ignatova et al. identified a subset of differentially translated transcripts that are enriched for genes involved in transcriptional regulation of different classes of RNA, according to GO analysis^16^. The loss of METTL5 did not change the abundance of 18S rRNA, suggesting that changes in translation are dependent on the methylation itself rather than ribosomal abundance or rRNA regulation^16^. However, this study also found a 15% reduction in global translation and a corresponding decrease in polysome abundance. The direct causal effect of m^6^A_1832_ on the translation efficiency of individual transcripts is unclear, and disentangling this from the broader alteration of feedback pathways that occurs in *METTL5* KO cells warrants additional research.

Next, Xing et al. used ribosome profiling sequencing (Ribo-seq) to identify 1,393 differentially translated gene transcripts (out of 9,093 actively transcribed genes), with 1,169 being downregulated in KO mESCs compared to WT mESCs^14^. FBXW7, an F-box protein that drives differentiation, was severely suppressed. WT METTL5, but not catalytically inactive METTL5, restored FBXW7 to WT levels, indicating that the methyltransferase activity of METTL5 directly drives the phenotype. Analysis of the mRNA transcripts that are translationally downregulated revealed a significant enrichment for A/T-rich codons, lower GC content in the coding sequence (CDS), and increased minimum free energy (MFE) in the CDS^14^. This work further supports the hypothesis that m^6^A_1832_ drives transcript-specific changes in translation and that which genes are affected is likely determined by the chemical and structural features of the individual mRNAs.

In contrast, Rong et al. found that both *METTL5* KO HeLa and *METTL5* KO HEK293T cells exhibit a global reduction in translation^18^. The KO cells show an increase in the number of 80 S monosomes and a decrease in polysome abundance, indicating that translation initiation may be impaired at a global scale^18^. This is in direct contrast with the findings in Chen et al. and Sepich-Poore et al., which showed no differences in monosomes or polysomes between *METTL5* KO and control HEK293T^15^ or HeLa^19^ cells. More research needs to be done to clarify these discrepancies and elucidate the molecular mechanisms of m^6^A_1832_ action in translation.

Sepich-Poore et al. found a slight growth defect in METTL5 KO HeLa and HepG2 cells. Despite the slower proliferation rate, their Ribo-seq analysis found no changes in global translation but rather a difference in the translation efficiency of a subset of transcripts involved in cell growth regulation. Because the growth defect was minor and global translation rates were not affected, the authors believe that the growth defect is not explained by METTL5 having a role in regulating ribosome biogenesis but possibly explained by a misregulation of a subset of genes involved in cell proliferation. More specifically, their Ribo-seq identified a significant downregulation of a calcium binding protein, CALM1, which is known to regulate cell proliferation. They also found an upregulation of genes that were associated with mitochondrial biogenesis and regulation, indicating an effect on cell metabolism. It is unclear whether this is a direct effect of the loss of m^6^A_1832_ or potentially a compensatory mechanism in response to the absence of METTL5. While this work is consistent with the studies described above, in that they identify transcript-specific consequences of METTL5 knockout, each of these studies has identified a unique subset of transcripts that are affected. This may be an artifact based on differential gene expression profiles across cell types or could point to a more context-specific function of m^6^A_1832_. A much more detailed understanding of the mechanism driving transcript-specific changes in translation is necessary to determine whether the studies performed to date are compatible.

## METTL5 in the brain and neural development

### Human genetic studies on METTL5 and intellectual disability

Although the molecular mechanism of m^6^A_1832_ is difficult to pinpoint, the broader phenotype of individuals with mutations in *METTL5* has been described in detail. A major finding that has driven interest in the study of *METTL5* is its connection to intellectual disability (ID) in humans. ID is a genetically heterogeneous disease characterized by significant limitations in cognitive function with disease onset before adulthood^46^. Mutations in *METTL5* were found to be associated with mild to severe forms of ID, with symptoms including muscular hypotonia, seizures, microcephaly, short stature, and malformations^11,12,40,47^. Richard et al. conducted GENCODYS^40^ and CARID^12^ analyses on two of the recent papers published on *mettl5* and human ID. This work identified biallelic frameshift variants in *mettl5* in two families of Pakistani and Yemenite origin with mild to severe forms of ID^13^. These individuals had normally functioning motor systems and no abnormalities in gait, hearing or vision but some behavioral effects, such as attention deficit hyperactivity disorder and aggressive behavior. The physical abnormalities included microcephaly, muscular hypotonia, short stature, low body weight, and abnormal dental morphology.

Upon further investigation of these *METTL5* mutations, Sanger sequencing revealed 2-bp deletions in family 1 (c.344_345delGA) and in family 2 (c.571_572delAA)^13^. The base pair deletion is predicted to cause premature truncation (p.Arg115Asnfs*19, p.Lys191Valfs*10) of the METTL5 protein. The c.344_345delGA mutation should hypothetically produce a protein that lacks the functional SAM domain and NPPF methyltransferase catalytic site, whereas those domains would not be affected by the c.571_572delAA mutation. Molecular modeling revealed that c.344_345delGA and c.571_572delAA are predicted to remove the evolutionarily conserved α-helix and/or β-sheets that make up the seven-β-fold in the C-terminal region of METTL5. The C-terminal residues compose the conserved binding region that interacts with SAM and allows the catalytic transfer of m^6^A_1832_ on the 18S rRNA^10^. If nonsense-mediated decay does not target the prematurely truncated *mettl5* transcript, it is likely that the protein is unstable or catalytically inactive.

**Fig. 4:**
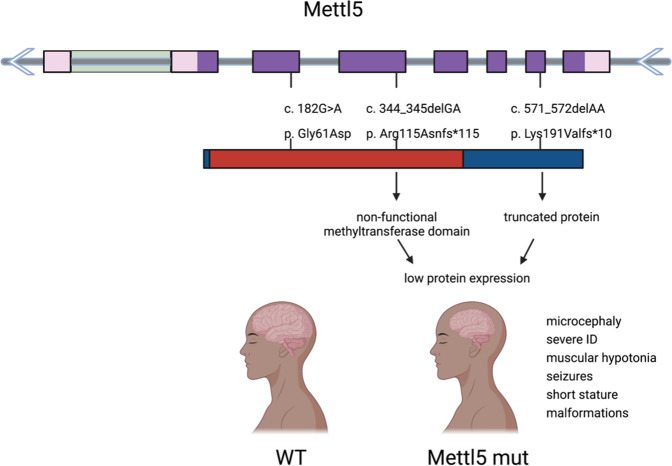
*METTL5* mutations cause protein truncation leading to a phenotype characterized by intellectual disability (ID) and microcephaly. The gene is shown with protein-coding exons in purple, non-transcribed exon regions in pink, and alternatively spliced regions in green. The protein is represented with the SAM methyltransferase domain in red and the rest of the protein in blue. The 2-bp deletions identified in humans cause early truncations of the protein that lead to the phenotype described in the *METTL5* mutant.

To better understand how these mutations affect METTL5 function within the cell, Richard et al. expressed three METTL5 mutant proteins—METTL5G61D, METTL5R115Nfs∗19, and METTL5L191Vfs∗10—in COS-7 cells and found that the proteins localized normally to the cytoplasm and nucleolus^13^ (Fig. 4). Even though the localization was normal, the protein levels of the frameshift mutations (METTL5R115Nfs∗19; METTL5L191Vfs∗10) were significantly reduced. When a proteinase inhibitor was added to these cells, the protein levels increased, indicating that the reduced levels were caused by protein instability. Future studies are needed to discern the functional impact of the METTL5G61D mutation as well as the mechanistic role that METTL5 plays in human brain development and intellectual disability.

### Translation regulation in neural cells

Previous work has shown that other METTL proteins are involved in self-renewal and pluripotency in humans and mice^48^. In the case of METTL5, there is conflicting evidence on whether *Mettl5* KO in mESCs affects self-renewal and pluripotency. Ignatova et al. found premature differentiation in naive *Mettl5* KO mESCs, accompanied by a decrease in the levels of pluripotency markers such as Nanog, Klf4, Sox2, and Rex1/Zfp42 and a parallel increase in levels of differentiation-associated markers^16^. Additionally, *Mettl5* KO mESCs showed impaired proliferation and aberrant apoptosis. This contrasts with the findings of Wang et al. and Xing et al., who found that naive *Mettl5* KO mESCs have no changes in proliferation rate or pluripotency markers^14,49^.

All three papers found an impairment in the KO mESC line’s ability to differentiate into all three germ layers (endoderm, mesoderm, and ectoderm), especially the neuroectoderm^14,16,49^. However, although differentiation was impaired, it was not completely abolished. Xing et al. found that after 6 weeks, teratomas eventually differentiated and had lineage-specific morphologies^14^. Upon further investigation, they found that METTL5 catalytic activity is required for timely differentiation, which indicates that m^6^A_1832_ may have a role in early differentiation, but the mechanism of m^6^A_1832_ activity was not explored. They proposed that the early differentiation effects may be due to a reduction in the translation of a protein involved in cell differentiation, FBXW7, in the *Mettl5* KO line^14^. When they conducted an FBXW7 knock-in in the *Mettl5* KO line, early differentiation was partially rescued, with a preference for neuroectodermal differentiation. It is not clear whether this regulation of early differentiation occurs through METTL5-mediated m^6^A_1832_ activity or a novel protein‒protein interaction impacted by its catalytic activity, since METTL5 has been proposed to have many RNA protein binding partners^15,18^. Despite the lack of clarity around the mechanism, METTL5 clearly has a significant role in early differentiation, especially for the neuroectoderm lineage, which further supports a role in brain development.

### Work in other model organisms

#### Mice

A 2016 study showed that SNPs in METTL5 were correlated with periodontitis in a mouse model of inflammation-mediated alveolar bone loss^50^. *Mettl5* attracted further interest as a regulator of neural activity when it was found to be ubiquitously expressed in the P30 mouse brain^13^. In 2020, multiple studies using *Mettl5* KO mice were published. These mice exhibit impaired survival (12.5%), reduced fertility, morphological abnormalities, and impaired memory and learning^16,19,49^. Similar to humans, KO mice have major craniofacial abnormalities with deviations in the nasal bone and incomplete fusion of the frontal bone^16,50^. Unlike humans, KO mice show eye size asymmetry and impaired hearing alongside degeneration of the seminiferous tubules of the testicles and increased spermatogonia debris in the epididymis^16^. Overall, these mice were less active and explored less than their wild-type counterparts^16^. Wang et al. similarly found that *Mettl5* KO mice were viable but much smaller in size than WT mice^19,49^. Both studies investigated the spatial learning abilities of these mice and found impaired learning and memory. RNA sequencing of the brains at P28 showed a downregulation of genes involved in neurodevelopment and myelin ensheathment^49^.

Sepich-Poore et al. confirmed that METTL5 KO mice were subviable and weighed <WT mice^19^. They also noted that the KO mice had reduced body fat compared to controls. Interestingly, RNA-seq of KO mouse brains and liver revealed a gene expression profile indicating defects in metabolism. More specifically, in the liver, there was a downregulation of genes involved in lipid metabolism and storage and an upregulation of the thyroid hormone responsive protein (Thrsp) gene, which could explain the reduction in body fat. In contrast with other studies, they did not find any defects in the learning or exploratory activity of these mice. This may be explained by their use of heterozygous KO mice as a control compared to the WT control used in the other studies^16,19,49^. Generally, the phenotypes seen in these mice recapitulate some of the phenotypes seen in humans, although exact quantification of intellectual disability in rodents is extremely difficult, and studies at the cellular and molecular levels are needed to identify conserved mechanisms of action.

#### C. elegans

In worms, metl-5, which has a coding sequence similarity of 93.5% to human *METTL5*^18^, was found to regulate stress responses specifically through the eicosanoid signaling pathway^33^. Eicosanoids are signaling lipids that mediate inflammation, tissue homeostasis, immunity, and cancer^51–55^. Metl-5 mutants are resistant to heat stress due to a reduction in eicosanoid synthesis. Rong et al. similarly found that metl-5 was important for stress responses^18^. The mechanism involves the unfolded protein response (UPR) in the endoplasmic reticulum (ER), although the details remain unknown.

Metl-5 mutants showed no differences in morphology, lifespan, or fertility compared to WT but did show increased survival under heat stress^33^ and decreased brood size^27^. In contrast, Rong et al. found that their metl-5 mutant did have an extended lifespan compared to WT; they also found no morphological abnormalities and increased survival under heat stress. In humans and mice, there are developmental effects, whereas stages of development occur normally in worms^18^.

At the molecular level, METL-5 is an 18S rRNA methyltransferase that adds an m^6^A at position A1717 in worm rRNA, and the 18S rRNA is methylated ~98% of the time^33^. Unlike mammals, METL-5 does not require any cofactors for its activity, and the TRMT112 ortholog C04H5.1 does not have an effect on METL-5 activity or m^6^A abundance on 18S rRNA^33^. METL-5 does not have an effect on global gene expression but rather impacts a small subset of transcripts, specifically those related to the eicosanoid synthesis pathway^33^. METL-5 may be involved in ribosomal biogenesis because a reduction in brood size has been associated with knockdown of ribosomal biogenesis-related proteins, but the mechanism has not been elucidated^27^. Knockdown of ribosomal protein gene transcripts generated lifespan phenotypes and stress resistance similar to those of the metl-5 mutant in the Rong et al. study, but the mechanism and the effect on translation warrant further investigation.

Unlike mammals, *C. elegans* have no other methyltransferases with the N6-adensoine catalytic motif, which suggests that METL-5 may be the only m^6^A methyltransferase in *C. elegans*^27^. Because METL-5 is the only modifier in worms, it may have alternate functions that separate it from its homologs in other species. In support of this idea, METL-5 does not require the cofactor TRMT112 for activation, which suggests that there may be slight differences in how it interacts with SAM and that it may even have an alternate binding partner.

#### Zebrafish

Richard et al. investigated the requirement for METTL5 in zebrafish development and found that zebrafish express a *METTL5* ortholog, *mettl5*, throughout development^13^. They used a specific translation blocking morpholino (MO) to knock down *mettl5* in embryos. At 72 h post-fertilization, they observed a reduction in the head size and tail curvature of the *mettl5* morphants, indicating developmental defects. Upon closer inspection of the brain, they found that the forebrain and midbrain were reduced in size, while the hindbrain was not significantly different. This finding reveals that *mettl5* has a role in zebrafish brain development, which complements the work related to intellectual disability in humans^13^. This finding highlights that METTL5 may be a fundamental protein involved in neural development across species. Future work needs to be done to confirm whether METTL5 has a similar mechanism as an 18S rRNA methyltransferase in zebrafish. This is likely, because zebrafish *mettl5* also contains an NPPF catalytic domain, and Mettl5 18S rRNA methyltransferase function is conserved across distantly related species^17,18,56^. Despite the microcephaly phenotype common in many Mettl5 KO/KD organisms, neuronal function has never been investigated in Mettl5 KD zebrafish. It is also unclear whether the zebrafish in this study survived to adulthood and if these zebrafish exhibited any behavioral effects, as seen in other species^13,16^. Future studies will need to provide more insight into the molecular and cellular effects of Mettl5 depletion in zebrafish development.

#### Drosophila

In *D. melanogaster*, Leismann et al. found the METTL5 ortholog Mettl5, which contains the conserved NPPF catalytic domain and has 53% sequence identity with human METTL5^17^. As expected, Mettl5 was found to be the m^6^A 18S rRNA methyltransferase, and almost all 18S rRNA was m^6^A modified. To investigate the function of Mettl5 in Drosophila, Liessman et al. created two Mettl5 mutants, one with a frameshift and the other with two conserved amino acids mutated. Both mutants were viable, fertile and had no visible morphological defects. Although both mutations should influence the secondary structure of the protein, only the frameshift mutation led to a lower abundance of m^6^A on 18S rRNA. Upon further investigation, they found that the Mettl5 frameshift mutation caused the flies to be severely disoriented, as determined by frequent changes in fly orientation and walking direction. This phenotype is supported by the finding that Mettl5 protein is expressed in all tissues but is highly enriched in the nervous system during Drosophila embryogenesis. In other species, METTL5 orthologs were found to be expressed in all tissues, but the expression pattern was not monitored over developmental time^18,39^. Interestingly, in Drosophila, a unique wave-like Mettl5 expression pattern across development was found. Mettl5 protein is highly expressed in the embryo, and its expression gradually decreases and remains low during the larval stage. It then increases again during metamorphosis and remains constant throughout adulthood. Future studies will need to be conducted to determine whether other species have a similar wave-like METTL5 expression pattern during development.

As previously stated, Mettl5 is expressed mainly in the cytoplasm of Drosophila cells, which contrasts with the METTL5 nucleolar localization found in the majority of mammalian cell lines^10,16,18^. Whether the localization and subsequent function of Mettl5 change over developmental time is an interesting question that could help explain some of the differential localization seen across species. Nevertheless, it is likely that regulation is similar between Drosophila and mammalian species, since Mettl5 heterodimerizes with the Trmt112 homolog, and the complex structure matches almost perfectly with the human METTL5-TRMT112 complex structure^17^. This indicates that the interaction with the SAM domain and the m^6^A modification on 18S rRNA is likely conserved, but the effect on translation or the timing of binding of 18S may differ from that in humans. In Drosophila embryonic S2R + cells, Trmt112 was found to be located predominantly in the nucleus with some localization in the cytoplasm. It is unclear how and when Mettl5 interacts with Trmt112, but Trmt112 is likely important for the 18S rRNA m6A modification, since the Mettl5-Trmt112 complex is conserved and Trmt112 depletion leads to lower amounts of m^6^A in total RNA, consistent with the m^6^A levels found in mettl5 mutants^17^. Similar to the case in mammals, ribosomal biogenesis was not affected in Drosophila Mettl5 mutants, indicating that Mettl5 is not necessary for rRNA processing but may be involved in altering translation efficiency. The authors propose that in flies, pre-ribosome-bound Mettl5 follows pre-40S ribosomes to the cytoplasm^17^. They further hypothesize that in human cells, METTL5 may dissociate from pre-40S in the nucleus^10,17^.

## Comparison of METTL5 and other m^6^A methyltransferases

The field of epitranscriptomics has been primarily focused on the m^6^A modification on mRNA that is installed by a METTL3/METTL14 heterodimer methyltransferase complex^57,58^. More recently, the functions of other Mettl family genes, such as Mettl5, have come to light. Although both add a methyl group onto adenine to form m^6^A, the differences between methylation on mRNA and rRNA are significant. First, m^6^A on mRNA is added to many different gene transcripts in a context-dependent manner and at various sites within the transcript^34^. In contrast, METTL5-installed m^6^A on rRNA is unique to a single nucleotide, A1832 on 18S rRNA^10^. The higher specificity of METTL5 compared to METTL3/METTL14 suggests a different mechanism of substrate selection, despite their similar RNA binding domains. In particular, both methyltransferase systems have important cofactors that likely direct their binding to RNA^10,59,60^. Since METTL family proteins have vastly different functions and substrates, the most direct comparison is in their structures.

The majority of METTL proteins, namely, METTL1, METTL2, METTL3, METTL5, METTL6, METTL14, and METTL15, are found in at least nine metazoan phyla and modify RNA^56^. METTL5 and several other METTL proteins (3,4,14,16, 25B) have similar functions in that they add an m^6^A modification to various forms of RNA. Interestingly, even though they have a shared function, these METTLs do not cluster together in the METTL phylogeny^56^. METTL5 clusters most closely to METTL16 in the METTL phylogeny (92% bootstrap)^56^, although METTL16 is still structurally distinct from METTL5^10^.

More specifically, there is a subset of methyltransferases that add m^6^A or m^6^A_m_ to various types of RNA. These include human *Mettl3/Mettl14*, *Mettl16, Capam, Mettl5, Zcchc4*, and *E. coli RlmJ*. The structures and substrates of these methyltransferases were very recently reviewed by Oerum et al*.*^21^. Briefly, all m^6^A methyltransferases have a Rossmann fold (a sequence of alternating beta strand (β) and alpha helical (α) segments in which the beta strands form a β-sheet via hydrogen bonds^61,62^) in their catalytic domains. Despite the low sequence identity across m^6^A methyltransferases, the root-mean-square deviation (RMSD: the average distance between atoms of superimposed proteins) is relatively low (~2.4–4.0 Å), indicating high structural similarity^21,63^. Interestingly, METTL3 has a catalytic motif distinct from those of METTL5 and most other m^6^A methyltransferases: METTL3 has a DPPW motif (Asp395-Pro398^METTL3^), whereas METTL16, CAPAM, and METTL5 have a NPPF catalytic motif^15^. Briefly, these motifs describe the amino acid makeup of the catalytic cavity in the methyltransferase and help regulate substrate binding^64^. Finally, the m^6^A methyltransferases with resolved structures all have three m^6^A methyltransferase loops around the active site pocket. These include an active site loop, a substrate binding loop, and a catalytic loop, which combinatorically contribute to RNA binding and enzyme catalysis. These structural similarities can be viewed in the figures of the review published by Oerum et al.^21^.

Beyond these structural similarities, the specificity of each methyltransferase depends largely on sequence or structure recognition. The METTL3-METTL14 methyltransferase complex has a known motif consisting of five nucleotides (DRACH) in single-stranded mRNA. In contrast, METTL16, ZCCHC4 and RlmJ all recognize a stem‒loop structure in RNA that is necessary for their substrate binding^21,65^. The METTL5-TRMT112 complex is highly specific for the methylation of A1832 in rRNA and actually has strong structural similarities to the DNA N6 methyltransferase M.Taql, although METTL5 is known to prefer single-stranded RNA, whereas M.Taql acts on double-stranded DNA^10,27^.

Moreover, the role of RNA methylation in development has become an interest in the field. Despite the high divergence of substrates and functions, m^6^A on mRNA and rRNA are both important in neural development^13,48^ and stem cell differentiation^16,66^. *Mettl5* KO mice survive longer than *Mettl3* KO mice, although both show defects in cognitive functions. In both cases, methylation of RNA seems to be a method of fine-tuning gene expression using posttranscriptional mechanisms.

## Future outlook

The field of epitranscriptomics is growing rapidly, in terms of both basic and translational science. The recent emergence of *METTL5* as another epitranscriptomic regulator of development and stem cell behavior reinforces the importance of posttranscriptional modifications as major regulators of cell behavior. One of the major difficulties moving forward will be untangling which of the hundreds of RNA modifications play regulatory roles in cell physiology and how they fit into the larger framework of transcriptional and translational regulation. Finally, the increased importance of RNA modifications in cognitive function, especially METTL5-mediated m^6^A modification of rRNA, highlights the need to understand the human-specific roles of the epitranscriptome. A combination of human genetics studies and careful modeling of human neural development will be necessary to unravel the complexities of the epitranscriptome in human biology.

Finally, a holistic view of the epitranscriptome that considers the many different modifications on multiple types of RNA is slowly starting to develop. Whether there is interplay among RNA modifications or more broadly between the epitranscriptome and other cell regulatory pathways will be an interesting avenue of research. As studies are performed on individual modifications, methyltransferases, protein readers, and other epitranscriptomic enzymes, researchers must incorporate the findings into the larger puzzle of the epitranscriptome. This will allow the field of epitranscriptomics to unearth whether and how the epitranscriptome functions as a basic regulatory system of gene expression across cell types and how it can be harnessed to mediate cell behavior in translational settings.
